# Assessment of medical professionalism: preliminary results of a qualitative study

**DOI:** 10.1186/s12909-020-1943-x

**Published:** 2020-01-30

**Authors:** Warren Fong, Yu Heng Kwan, Sungwon Yoon, Jie Kie Phang, Julian Thumboo, Ying Ying Leung, Swee Cheng Ng

**Affiliations:** 1 Department of Rheumatology and Immunology, Singapore General Hospital, Singapore, Singapore; 2 Duke-NUS Medical School, Singapore, Singapore; 3 NUS Yong Loo Lin School of Medicine, National University of Singapore, Singapore, Singapore; 4 SingHealth Rheumatology Senior Residency Programme, 20 College Road, Singapore, 169856 Singapore; 5 Program in Health Services and Systems Research, Duke-NUS Medical School, Singapore, Singapore

**Keywords:** Professionalism, Singapore, Qualitative, Assessment

## Abstract

**Background:**

The importance of medical professionalism has been well documented in the literature. Cultural background affects the constituents of professionalism. However, few studies have explored the domains of medical professionalism in an Asian context. We aim to describe the views and experiences of both patients and faculty on medical professionalism in an urban Asian city state.

**Methods:**

Data for this qualitative study were collected through focus group discussions (FGDs) with patients and in-depth interviews (IDIs) with faculty members. The IDIs and FGDs were audio-recorded and transcribed verbatim. Thematic analysis was conducted by two independent coders using a priori framework derived from Professionalism Mini Evaluation Exercise (P-MEX). Sociodemographic data of faculty members and patients were obtained through brief questionnaires.

**Results:**

A total of 31 patients (58.1% male, all had visited healthcare facilities within the past year) and 26 faculty members (42.3% male, 38.5% from medical disciplines, median years as faculty is 11) participated in this study. The results supported the four domains of medical professionalism - Doctor-patient relationship skills, Reflective skills, Time management and Inter-professional relationship skills. Two new sub-domains which emerged from data were (1) communicated effectively with patient and (2) demonstrated collegiality.

**Conclusions:**

The domains of professionalism in Singapore were similar to previous studies. This study allows a better understanding of medical professionalism to enhance the assessment and ultimately, the teaching of medical professionalism in an urban multi-ethnic Asian city.

## Background

In recent years, medical professionalism has been increasingly emphasized in medical undergraduate and post-graduate curricula [1–4]. Previously ‘good medical practice’ has been defined more broadly in terms of roles such as ‘professional’ and ‘manager’, with various medical councils, academic and professional bodies having produced clear documentations on these roles [5–7]. In 2002, a set of professional attributes was defined in the Physician’s charter on Medical Professionalism, defining a professional doctor as having professional competence, being honest with patients, maintaining patients’ confidentiality, maintaining appropriate relationships with patients, being able to improve quality of care, ensuring just distribution of resources, possessing scientific knowledge, being able to manage conflicts of interest and possessing professional responsibilities [1]. The charter is overseen by leaders in the American Board of Internal Medicine (ABIM) Foundation, the American College of Physicians-American Society of Internal Medicine (ACP–ASIM) Foundation, and the European Federation of Internal Medicine [1]. To date, the charter has been endorsed by 108 national and international organizations [8]. Medical professionalism has been shown to affect doctors’ relationships with their patients, quality of care, and ultimately health and illness outcomes [9]. For residents in training, unprofessional behaviour during their training resulted in increased risk of disciplinary action later on in their careers as physicians [10]. Fortunately, medical professionalism can be nurtured [11]. Thus, assessment of medical professionalism in daily practice of trainees becomes increasingly important as part of their development of their professional identity, with the aim of being able to provide timely feedback and early remediation [12].

There have been studies attempting to define the domains of professionalism using qualitative methods [13–16]. Wagner et al. has identified knowledge/technical skills, patient relationship and character virtues as main themes of professionalism [13], while Jha et al. has identified compliance to values, patient access, doctor-patient relationship, demeanour, professional management, personal awareness and motivation as the themes of professionalism [14]. However, none of these studies were performed in the context of Asian medical education. As highlighted by the cultural value theory and cultural dimension theory, cultural differences exist between countries [17, 18]. Previous study has shown that the conceptualization of professionalism is influenced by culture [19–21]. For example, altruism was found to be an essential element of medical professionalism for Asia and North America, but not Europe [21]. In addition, the constituents of medical professionalism in China was influenced by its longstanding Confucian traditions [22].

We aim to describe the views and experiences of both patients and faculty on medical professionalism in an urban multi-ethnic Asian city. In doing so, we hope to be able to improve our understanding of medical professionalism in Asia and to develop culturally-adapted tools to measure and improve medical professionalism.

## Methods

### Study design

We used semi-structured interviews to elicit participants’ views and experiences of medical professionalism from December 2017 to October 2018. We anchored our methodology according to the Consolidated Criteria for Reporting Qualitative Research (COREQ) checklist (Supplementary data) [23]. The following purposive sampling technique was performed to provide a range of views on medical professionalism: recipients of healthcare services were selected based on age, gender, ethnicity, education and socio-economic status while medical educators responsible for medical training and assessment were selected according to age, gender, ethnicity and disciplines for faculty in SingHealth residency. SingHealth is Singapore’s largest group of public healthcare institutions, consisting of four public hospitals, five national specialty centres and a network of community hospitals and polyclinics [24]. There are over 1600 faculty and more than 900 residents in training, with yearly outpatient attendances of about 4 million. As participants accepted the invitations and scheduled interviews, we iteratively adjusted our subsequent invitations to ensure a reasonably balanced representation of recipients of healthcare services and medical educators. Some participants might be colleagues or patients of the researchers (WF, JT, YYL and SCN). However, these researchers were not involved in the recruitment and interview processes to minimise potential influence on the participants.

### Focus group discussions (FGDs) with patients

We invited patients who had received outpatient and/or inpatient care in any healthcare facilities to participate in FGDs through telephone and email after referral from attending doctors in different disciplines. One patient refused to participate due to discomfort with the interview. We conducted FGDs with patients in English or Mandarin in a quiet room. Each FGD lasted approximately 75 to 90 min. All FGDs were facilitated by a moderator, in the presence of another study team member who served as an observer and a note taker. The moderators had no prior relationship with the participants prior to study commencement. During session introductions, interviewers shared only their name, job title, and role in the project with study participants.

Moderators used a standardized guide (see supplementary materials) to identify issues related to medical professionalism that were important to patients. Our systematic review had earlier indicated that P-MEX was one of the promising tools to assess medical professionalism [25]. Therefore, we developed our topic guide based on the domains and items of the P-MEX, with an intent to adapt this tool for use in Singapore. The original P-MEX consists of 4 domains (Doctor-patient relationship skills, Reflective skills, Time management and Inter-professional relationship skills) and 21 sub-domains [26]. We started with questions that were open-ended, with some prompts to ensure consistency and coverage of topics across groups. The interview guide was pilot-tested. We divided the FGD into 2 parts. First, patients were asked to list the traits they considered important for professionalism in all doctors. Patients were then asked to discuss these traits in the group discussion. Next, patients were asked to discuss the 10 items from the Doctor-patient relationship and Reflective skills domains of P-MEX in terms of assessing demeanours of a professional doctor in the local cultural and healthcare context. The patients were also invited to list any missing item which may be important for the assessment of a professional doctor. Focus groups were conducted until data saturation was reached.

### In-depth interviews (IDIs) with medical faculty

As it was difficult to coordinate the schedules among faculty members, we conducted semi-structured IDIs with faculty members in English in a quiet room. Each IDI lasted approximately 30–45 min. All IDIs were facilitated by the same moderator as the FGD. The standardized guide was similar to the one used for FGD, but for the second part, faculty members were asked if there were any items that they felt were not relevant, and to choose up to five least relevant items in assessing medical professionalism if applicable. The faculty were also invited to list any missing item which may be important for the assessment of a professional doctor. Interviews were conducted until data saturation was achieved.

### Data analysis

FGDs and IDIs were voice-recorded and transcribed verbatim. Thematic data analysis was conducted by two independent coders (YHK and JKP), who were both trained in qualitative research, to ensure inter-coder reliability. The method of analysis chosen for this study was a hybrid approach of qualitative methods of thematic analysis, and it incorporated both the inductive approach based on the grounded theory and the deductive approach using a priori template of codes [27, 28]. Elements of grounded theory was adopted in the analysis of the data, allowing new codes and categories outside of the P-MEX to emerge during the analysis of each transcript, encouraging the development of a conceptual framework from the input of participants. For example, when provision of clear and honest information was discussed extensively by participants, it was selected as one open coding category, positioning it as a central category of the indicators. Provided clear and honest information was subsequently recoded into communicated effectively with patient (axial coding) when similar categories emerged from the data such as communicated empathetically. We used NVivo 11 software to facilitate the data analysis process.

The four domains of medical professionalism from the P-MEX (Doctor-patient relationship skills, Reflective skills, Time management and Inter-professional relationship skills) were used as a priori coding template to support the analysis [26]. The initial cycle of coding was done by deductive approach using a priori template of codes, and the second cycle of coding was done to identify any new domains or sub-domains using inductive approach. The primary researchers (YHK and JKP) discussed each stage of the analysis with the research team (WF, SY, YYL, SCN). Discrepancies in interpretation of materials were resolved through an iterative discussion amongst research team members until a list of codes that could be consistently applied was compiled.

In order to maintain the methodological rigour of qualitative research, the following strategies were systematically applied to our study based on the Lincoln and Guba’s four criteria [29]: credibility was achieved through pilot testing of the topic guides and collection and inclusion of field notes for data analysis. We have also reported preliminary findings at several scientific meetings to gain insights and views from relevant parties; dependability was achieved by a detailed track record of the data collection process and assessment of coding accuracy and inter-coder agreement among the research team throughout the analysis process; confirmability was achieved through data triangulation (i.e. interviews with both patients and faculty); and transferability was achieved through purposive sampling to ensure that the selected participants were representative of the views of patients and faculty. We also employed iterative interpretations of findings until no new codes emerged from the dataset and all variations in key concepts were identified.

### Ethics

The SingHealth Centralized Institutional Review Board approved this study (Ref No: 2016/3009). We obtained informed consent, which conformed to the principle outlined in the 1964 Declaration of Helsinki, from all patients and faculty before the interview.

## Results

A total of 31 patients (58.1% male, median age 32 years old, age range from 22 to 75 years old) participated in 6 FGDs (4 conducted in English and 2 conducted in Mandarin) while a total of 26 faculty members (42.3% male, median age 42 years old, age range from 26 to 76 years old) participated in the IDIs (all conducted in English). All patients had visited healthcare facilities within the past year (Table 1). Based on the data from 2018 from the Department of Statistics Singapore [30], the majority of the residents are Chinese (74%), followed by Malays (13%) and Indians (9%). The percentage of residents with post-secondary education is 55.8%. The median age of the population is 40.8 years. One-third of the faculty members came from medical disciplines, the rest spread across a wide spectrum of disciplines (anesthesiology, surgical, diagnostic radiology, nuclear medicine and pathology, emergency medicine, paediatrics, surgical and allied health). The FGDs ranged in size from 4 to 6 participants. Data saturation occurred after 18 IDIs and 5 FGDs, with no new themes emerging. The socio-demographic characteristics of the participants in the FGD and IDI are summarized in Tables 1 and 2.

**Table 1:** Demographics profile of patients who participated in focus group discussions (*n* = 31)

Characteristics	Number (%)
Age	
21–29	12 (38.7)
30–39	6 (19.4)
40–49	1 (3.2)
50–59	6 (19.4)
60–69	4 (12.9)
70–79	2 (6.5)
Gender	
Male	18 (58.1)
Female	13 (41.9)
Ethnicity	
Chinese	27 (87.1)
Malay	2 (6.5)
Indian	2 (6.5)
Highest education attained	
Secondary and below	5 (16.1)
Post- secondary	26 (83.9)
Marital status	
Single	18 (58.1)
Married	13 (41.9)
Employment status	
Employed	18 (58.1)
Unemployed	13 (41.9)
Housing	
Public housing	18 (58.0)
Private housing	11 (35.5)
Hostel	2 (6.5)
Healthcare facility visited in the past 1 year	
Polyclinic	18 (58.1)
General practitioner	20 (64.5)
Hospital admission	6 (19.4)
Specialist outpatient clinic	13 (41.9)

**Table 2:** Demographics profile of faculty members who participated in interviews (*n* = 26)

Characteristics	Number (%)
Age	
21–29	2 (7.7)
30–39	8 (30.8)
40–49	11 (42.3)
50–59	3 (11.5)
60–69	0 (0)
70–79	2 (7.7)
Gender	
Male	11 (42.3)
Female	15 (57.7)
Years as faculty	
2–10	13 (50.0)
> 10	13 (50.0)
Ethnicity	
Chinese	17 (65.4)
Malay	1 (3.8)
Indian	8 (30.8)
Disciplines	
Medical disciplines ^a^	10 (38.5)
Anesthesiology	2 (7.7)
Diagnostic radiology, Nuclear Medicine and Pathology	2 (7.7)
Emergency medicine	1 (3.8)
Paediatrics	2 (7.7)
Surgical disciplines ^b^	3 (11.5)
Allied Health ^c^	6 (23.1)

^a^ includes respiratory medicine, dermatology, neurology, nephrology, internal medicine, infectious disease

^b^ includes general surgery, obstetrics and gynaecology

^c^ includes pharmacy, physiotherapy, medical social service, podiatry, nursing, and occupational therapy

### Framework of medical professionalism

**Fig. 1:**
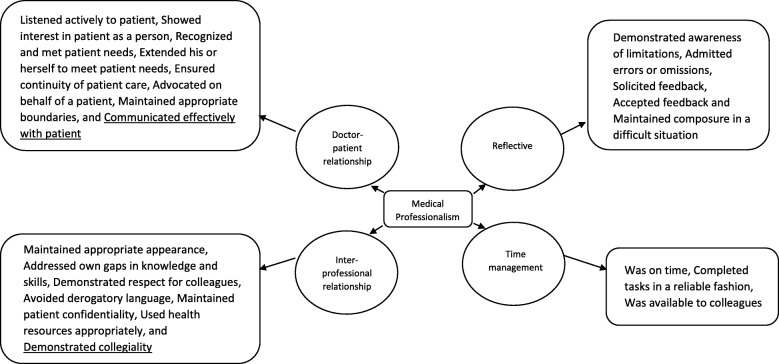
Domains and subdomains of medical professionalism. Underlined sub-domains are the new sub-domains derived from this study

The framework for medical professionalism for this study is shown in Fig. 1. Using the a priori framework for medical professionalism of P-MEX, 4 domains were derived (Doctor-patient relationship skills, Reflective skills, Time management and Inter-professional relationship skills). There were 21 codes which were mapped against the P-MEX. Three new codes emerged from the data. These codes were subsequently merged into 23 sub-domains and 4 domains (Table 3).

**Table 3:** Representative quotes from interviews with faculty and patients

Sub-domain	Faculty	Relevance	Patient	Relevance
	Quotation		Quotation	
Domain: Doctor-patient relationship skills				
Listened actively to patient	Professional doctors must be good listeners, the opportunity for the family or the patient to get things off their chest is very important. *– Faculty, 38 years old, Female, Indian*	+	As a doctor, I feel that you should not cut the patient short. - *Patient, 23 years old, Male, Chinese*	+
Showed interest in patient as a person	A professional doctor should not treat the disease only but treat the patient as a whole, and look at the patient as a whole, as a person, as a person like a family in the community. - *Faculty, 50 years old, Male, Chinese*	+	Rather than just seeing the patients as just someone [who needs to be] diagnosed, it is important to see them as individuals who have emotions. *- Patient, 23 years old, Female, Indian*	+
Recognized and met patient needs	A professional doctor needs to find from the patient what they want, how can we help them overcome the disease condition as much as possible. - *Faculty, 50 years old, Male, Chinese*	+	Doctors should listen to what the patients want, not [just provide] what they want for the patients. - *Patient, 30 years old, Female, Chinese*	+
Extended his/herself to meet patient needs	[When parents refused admission], we offered alternative [transfer to another institution], and when they didn’t accept that, our next step was [getting] social worker to visit, speak to the parents, see how they could help. - *Faculty, 43 years old, Male, Indian*	+	Doctors do go beyond what they are supposed to do. They contact social services to help arrange for patients to make sure that they are compliant to their medications, because otherwise there is no point in treating them if they are not compliant. - *Patient, 28 years old, Female, Chinese*	+
	Going above and beyond, I think it’s just icing on the cake. I think also has to be taken in context because sometimes patients or family may have needs that are beyond our ability to actually fulfil, or sometimes it may not actually be appropriate. -*Faculty, 37 years old, Female, Chinese*	–	Everyone is really busy and only have certain amount of time with the patient, I think that as long as you do your job, like you try to understand the patient and meet the patient needs, that’s good enough. -*Patient, 30 years old, Female, Chinese*	–
Ensured continuity of patient care	Professional doctor will refer patients to relevant colleagues in the other specialty. - *Faculty, 50 years old, Male, Chinese*	+	Doctor can refer patients to specialist in that field. - *Patient, 24 years old, Female, Chinese*	+
Advocated on behalf of a patient	If the patient needs financial assistance, a doctor should know about that, and initiate referral. - *Faculty, 42 years old, Female, Chinese*	+	The doctor advocated for my mother to get some form of subsidy because it’s a very expensive surgery … I think she stayed for 1 month in the ICU, so the cost is very high. It’s actually quite heartening that the doctor care about your financial concern, not just about performing the surgery and saving your life. - *Patient, 30 years old, Male, Chinese*	+
	Healthcare professionals should try to advocate patients to help their own, not just rely on the healthcare professionals, the doctors. *- Faculty, 27 years old, Female, Chinese*	–	Not applicable	
Maintained appropriate boundaries	Doctors need to be sensitive to religion and culture, and not say something or do something which will be culturally taboo. - *Faculty, 49 years old, Male, Chinese*	+	A professional doctor maybe [mean] keeping things professional, keeping a distance from the patients unless necessary. - *Patient, 24 years old, Female, Chinese*	+
Communicates effectively with patient	Trying to explain the clinical scenario, their problems, whatever treatment they undergo, and at the same time, answering their questions, taking them into confidence, making sure that they understand, checking their understanding. - *Faculty, 49 years old, Female, Indian*	+	When doctors are explaining the patient’s medical condition, they should use a more layman and general term, to ensure that the patient understand. A professional doctor should stand in the shoes of the patient when explaining the patient’s condition, instead of just bombarding all the medical terms, which some patients may not even understand. - *Patient, 24 years old, Female, Chinese*	+
Domain: Reflective skills				
Demonstrated awareness of limitations	Trying to do something that you don’t understand is unprofessional. Not asking for help at the right time, not knowing your limits, not knowing the limits of your knowledge and skills is also unprofessional behaviour. *- Faculty, 49 years old, Female, Indian*	+	If you are not able to do it, you probably think of referring it to somebody or you talk to somebody. - *Patient, 60 years old, Male, Chinese*	+
Admitted errors/omissions	Sometimes during emergency resuscitation situation, there may be lapses. Doctors really have to be brave enough to own up to that mistake, because if you don’t, you may not get corrective action done. - *Faculty, 43 years old, Male, Indian*	+	Professional means you must be able to own up to the mistake you make. - *Patient, 32 years old, Female, Malay*	+
Solicited feedback	Sometimes there are things that doctors don’t realize so it’s good to get feedback from others to improve yourself. - *Faculty, 26 years old, Female, Indian*	+	A doctor needs to listen to other people’s opinions, to reflect whether or not he has this ability to meet the requirements. I think he must listen to others before he can know whether he has met the requirements of a doctor. - *Patient, 55 years old, Female, Chinese*	+
	If you are a junior doctor, feedback will be given to you, you don’t have to look for it. The faculty are forced to fill up feedback form for them. *-Faculty, 34 years old, Male, Malay*	–	The doctor doesn’t need to solicit feedback actively like ask every single patient whether you have any feedback, any way I can improve myself as a doctor? I feel it is unnecessary. *-Patient, 24 years old, Female, Chinese*	–
Accepted feedback	It’s important to have the humility, to accept people’s feedback, and try to reflect. *- Faculty, 34 years old, Female, Chinese*	+	It’s key in the doctor profession, to take feedback, learn from it, and become better. - *Patient, 32 years old, Female, Malay*	+
Maintained composure in a difficult situation	If a doctor gets angry because the patient is getting angry, then the doctor is not professional. - *Faculty, 38 years old, Female, Indian*	+	Sometimes doctors have to meet some unreasonable patients, or emotional patients, and they have to endure their bad emotions. - *Patient, 70 years old, Female, Chinese*	+
Domain: Time management				
Was on time	When you are punctual, you are not only respecting yourself, but also the person you are dealing with, for example the patients or sometimes even the colleagues when we are having meeting. *- Faculty, 58 years old, Male, Chinese*	+	I feel doctors should be punctual. - *Patient, 52 years old, Female, Chinese*	+
Completed tasks in a reliable fashion	The junior doctors have assignments to do, assessments to do, reports to fill. All these should be done in the time allotted to it. *- Faculty, 46 years old, Male, Indian*	+	Not applicable	
Was available to colleagues	A professional doctor will take into account team members’ welfare as well. When we work in a team, we make sure that people are not over-worked, and help colleagues if they need our help. *- Faculty, 38 years old, Male, Chinese*	+	Not applicable	
	Only doctors who have done very well have spare capacity to help other colleagues. If they are not good enough, sometimes it is not unprofessional, it is just that they do not have the capacity to help others. *- Faculty, 58 years old, Male, Chinese*	–	Not applicable	
Domain: Inter-professional relationship skills				
Maintained appropriate appearance	Society has got some norms about how they expect doctors to look like. If you dress outside those norms, and because you dress like that make the patients distrust you or worse still distrust the doctors in general, then that is inappropriate. - *Faculty, 46 years old, Male, Indian*	+	We would want to see a doctor who is clean and decent. - *Patient, 35 years old, Female, Chinese*	+
Addressed own gaps in knowledge and skills	Professional doctor has to show that he has been in keeping up with the technology updates in medicine through on-going continuing medical education courses, go to conferences for updates. - *Faculty, 49 years old, Female, Chinese*	+	Professional doctors need to know what are the latest technological advances to help patients better. - *Patient, 28 years old, Female, Chinese*	+
Demonstrated respect for colleagues	We should take other healthcare professionals’ opinion respectfully, and consider whether the opinion is correct, or whether they are of certain merit, rather than discount them totally. *- Faculty, 38 years old, Male, Chinese*	+	Not applicable	
Avoided derogatory language	I mean the comments or feedback should be made as objective as possible. You just highlight what your colleague has done wrong and give them advice, rather than saying, “You are lazy” or “You cut corners” and all that. *- Faculty, 38 years old, Male, Chinese*	+	Not applicable	
Maintained patient confidentiality	Confidentiality is of course avery important part of doctor-patient relationship. Not divulging patient’s medical information on social media. *- Faculty, 38 years old, Male, Chinese*	+	Not applicable	
Used health resources appropriately	Making the best use of limited resources to give the patients the best possible things which are consistent with their values would be an independent skill of professionalism. - *Faculty, 46 years old, Male, Indian*	+	Not applicable	
	Sometimes certain buffers need to be put in place because unexpected things can happen. I rather be safe and use slightly more resources than to be sorry especially when something unexpected happen. *- Faculty, 50 years old, Male, Chinese*	–	Not applicable	
Demonstrated collegiality	Giving other colleagues responsibility, so certain departments do not give nurses much responsibilities, even though they have master’s degree in nursing. *- Faculty, 49 years old, Male, Chinese*	+	Not applicable	

+ represents relevant

- represents not relevant

### Doctor-patient relationship

Our data mapped against all 7 original sub-domains in doctor-patient relationship, namely (1) listened actively to patient, (2) showed interest in patient as a person, (3) recognized and met patient needs, (4) extended his or herself to meet patient needs, (5) ensured continuity of patient care, (6) advocated on behalf of a patient, (7) maintained appropriate boundaries. However, the sub-domain “extended his or herself to meet patient needs” was deemed to be less relevant by both faculty members and patients. They felt that extending oneself to meet the needs of the patient may be impractical, as some needs may be beyond the doctor’s ability to fulfil. The faculty and patients felt that it would be sufficient if a doctor could fulfil the requirements of other sub-domains, as shown in the following quote “I think what is needed here is just the doctor to actively listen. I think that’s enough, because to extend the extra effort, the doctor may not have enough time.” *(Patient, 22 years old, Male, Chinese).*

We also found a new sub-domain, “communicated effectively with patient”. It was repeatedly highlighted by patients that it was important for a professional doctor to take time to explain the disease, treatment options, risks and benefits, and ensure that patients understood. Faculty also stressed the importance of communication skills for a professional doctor.

### Reflective skills

Our data mapped against all 5 original sub-domains in reflective skills, namely (1) demonstrated awareness of limitations, (2) admitted errors or omissions, (3) solicited feedback, (4) accepted feedback and (5) maintained composure in a difficult situation.

The sub-domain “solicited feedback” was deemed to be less relevant by faculty members. Faculty members commented that feedback will be given to trainees, therefore it is not necessary for the trainees to seek feedback actively. Patients also felt uncomfortable if doctors solicited feedback from them, as evident from the following quote: “It makes me completely lose confidence if the doctor ask me how he/she is doing.” *(Patient, 75 years old, Female, Chinese).*

### Time management

Our data mapped against all 3 original sub-domains in time management, namely namely (1) was on time, (2) completed tasks in a reliable fashion, (3) was available to colleagues.

The faculty clarified that while respect for other people’s time was important and doctors should strive to be punctual, there are circumstances where doctors can be delayed, for example, medical emergencies and complicated patients who requires a longer time to manage.

### Inter-professional relationship

Our data mapped against all 6 original sub-domains in inter-professional relationship, namely (1) maintained appropriate appearance, (2) addressed own gaps in knowledge and skills, (3) demonstrated respect for colleagues, (4) avoided derogatory language, (5) maintained patient confidentiality, (6) used health resources appropriately. We also found a new sub-domain, “demonstrated collegiality”. Faculty stressed the importance of teamwork in medicine, where it was important to respect the contribution of each healthcare worker in the care of the patient, and cooperating with one another to ensure the best outcome in the care of the patient. This is evident in the following quote, “Medical care for the patient is a teamwork, because doctors can make all the orders, but you need the nurses to carry out the orders and of course you need a lot of help from allied health professionals, like physiotherapists, occupational therapists, dieticians, speech therapists.” *(Faculty, 32 years old, Female, Chinese).*

Faculty members did not completely agree as to what is “appropriate use of health resources”. Some faculty members felt that overutilization or underutilization of health resources such as hospital beds, expensive drugs and scans, may be due to other reasons such patient’s financial resources and personal convictions. For example, faculty mentioned that patients who had the financial resources might request for more investigations than needed.

### Least relevant sub-domains as deemed by faculty

The top three least relevant sub-domains as chosen by the faculty were “solicited feedback” (*n* = 17), “extended his or herself to meet patient needs” (*n* = 16), “used health resources appropriately” (*n* = 15). As mentioned previously, the faculty members commented that feedback will be given to trainees routinely, therefore it is not necessary for the trainees to seek feedback actively. The faculty also felt that extending oneself to meet the needs of the patient may be impractical. The faculty also commented that the inappropriate use of health resources by trainees may “not be because they were unprofessional but due to inexperience” *(Faculty, 32 years old, Female, Chinese).*

## Discussion

The results from our current study supported the four domains of medical professionalism derived from the P-MEX [26], namely Doctor-patient relationship skills, Reflective skills, Time management and Inter-professional relationship skills. In addition, 2 new sub-domains “communicated effectively with patient” and “demonstrated collegiality” under the domains of Doctor-patient relationship skills and Inter-professional relationship were identified respectively. The least relevant sub-domains as chosen by the faculty were “solicited feedback”, “extended his or herself to meet patient needs”, “used health resources appropriately”.

The four domains of medical professionalism identified in this study also are similar to the components of medical professionalism as highlighted by the General Medical Council, namely “Behave according to ethical and legal principles”, “Reflect, learn and teach others”, “Learn and work effectively within a multi-professional team”, “Protect patients and improve care” [7]. Previous studies have identified knowledge/technical skills, patient relationship, character virtues, compliance to values, patient access, doctor-patient relationship, demeanour, professional management, personal awareness and motivation as main themes of professionalism [13, 14]. These themes of professionalism can be found in domains and sub-domains of professionalism in this study.

Our study has also identified new sub-domains not present in the original P-MEX. Under the Doctor-patient relationship skills domain, the new sub-domain is “communicated effectively with patient”. The emergence of the new sub-domain “communicated effectively with patient” illustrates the shift in medical care towards one with emphasis on patient autonomy [31]. Traditionally, doctor-patient relationships were shaped by paternalism where doctors had a high degree of control over the patients, and this was more apparent in Asian context [32]. However, the communication model is moving towards more egalitarian partnership, with greater awareness of medical consumerism [33]. The importance of communication skills in medical professionalism has also been highlighted in other studies [19, 21]. Under the Inter-professional relationship skills domain, we identified a new sub-domain “demonstrated collegiality”. According to Hofstede’s cultural dimension theory, Asians tend to be more collectivist compared to Western populations [18], and this was highlighted in the new sub-domain “demonstrated collegiality”, which demonstrates the faculty’s perception of the increasing importance of team-based care and collaboration between various healthcare professionals, whose opinions should be respected [34, 35]. The importance of collegiality was also highlighted in the study by Chandratilake et al. who showed that working with one’s colleagues towards common goals was deemed to be important in European and North American countries [21].

The strengths of this study include purposive sampling to ensure that a broad range of views about medical professionalism elicited from both patients and faculty. The patients recruited for this study mirrored the ethnicity distribution of the general population in Singapore [36]. In addition, we included patients of different age groups and patients attending primary care as well as tertiary care institutions in the FGDs. We also ensured that faculty members across different disciplines were included in the IDIs. To the best of our knowledge, this is the first qualitative study performed in Asia to explore medical professionalism.

Limitations of this study include limited generalizability of findings to other Asian countries as this study was done in a single Asian country, and perception of medical professionalism is affected by different cultural context. However, since there few studies exploring medical professionalism in Asia, this study provides a basis for conducting future research on medical professionalism, especially in Asia. Secondly, patients who were purely Malay and Tamil speakers were not included. However, the impact of this on the results is likely to be small, given that pure Malay and Tamil speakers only formed 1.2 and 0.29% of the resident population in 2015 [37]. In addition, the coding frame was based on the domains of medical professionalism from P-MEX. Therefore, we may have missed certain attributes of medical professionalism that were not included in the P-MEX. However, the qualitative nature of our study provided a nuanced understanding of medical professionalism within the context of an Asian healthcare setting, identifying two new sub-domains. The percentage of residents with post-secondary education was 84% in our study, which was higher than that of the general population (55.8%). However, this is not likely to have an impact as difference in perception of professionalism for patients with different education level was not observed for our study and other study as well [38].

## Conclusion

In conclusion, we found that Doctor-patient relationship skills, Reflective skills, Time management and Inter-professional relationship skills are relevant to both faculty members and patients as domains of medical professionalism. New sub-domains such as “communicating effectively with the patient” and “demonstrated collegiality” were also found to be important to medical professionalism in Singapore. Future research in Asian countries may consider including these sub-domains for assessment of medical professionalism.

## Data Availability

The datasets used and/or analysed during the current study are available from the corresponding author on reasonable request.

## Abbreviations

ABIM: American Board of Internal Medicine
ACP–ASIM: American College of Physicians-American Society of Internal Medicine
COREQ: Consolidated Criteria for Reporting Qualitative Research
FGD: Focus group discussion
IDI: In-depth interviews
P-MEX: Professionalism Mini Evaluation Exercise
